# Development and Validation of Decision Rules Models to Stratify Coronary Artery Disease, Diabetes, and Hypertension Risk in Preventive Care: Cohort Study of Returning UK Biobank Participants

**DOI:** 10.3390/jpm11121322

**Published:** 2021-12-07

**Authors:** José Castela Forte, Pytrik Folkertsma, Rahul Gannamani, Sridhar Kumaraswamy, Sarah Mount, Tom J. de Koning, Sipko van Dam, Bruce H. R. Wolffenbuttel

**Affiliations:** 1 Department of Clinical Pharmacy and Pharmacology, University Medical Center Groningen, University of Groningen, Hanzeplein 1, 9713 GZ Groningen, The Netherlands; 2 Ancora Health B.V., Herestraat 106, 9711 LM Groningen, The Netherlands; pytrik@ancora.health (P.F.); rahul@ancora.health (R.G.); sridhar@ancora.health (S.K.); sarah@ancora.health (S.M.); sipko@ancora.health (S.v.D.); 3 Department of Endocrinology, University Medical Center Groningen, University of Groningen, Hanzeplein 1, 9713 GZ Groningen, The Netherlands; bwo@umcg.nl; 4 Department of Neurology, University Medical Center Groningen, University of Groningen, Hanzeplein 1, 9713 GZ Groningen, The Netherlands; tom.j_de_koning@med.lu.se; 5 Pediatrics, Department of Clinical Sciences, Lund University, Sölvegatan 19-BMC F12, 221 84 Lund, Sweden

**Keywords:** coronary artery disease, hypertension, diabetes, preventive medicine, risk stratification

## Abstract

Many predictive models exist that predict risk of common cardiometabolic conditions. However, a vast majority of these models do not include genetic risk scores and do not distinguish between clinical risk requiring medical or pharmacological interventions and pre-clinical risk, where lifestyle interventions could be first-choice therapy. In this study, we developed, validated, and compared the performance of three decision rule algorithms including biomarkers, physical measurements, and genetic risk scores for incident coronary artery disease (CAD), diabetes (T2D), and hypertension against commonly used clinical risk scores in 60,782 UK Biobank participants. The rules models were tested for an association with incident CAD, T2D, and hypertension, and hazard ratios (with 95% confidence interval) were calculated from survival models. Model performance was assessed using the area under the receiver operating characteristic curve (AUROC), and Net Reclassification Index (NRI). The higher risk group in the decision rules model had a 40-, 40.9-, and 21.6-fold increased risk of CAD, T2D, and hypertension, respectively (*p* < 0.001 for all). Risk increased significantly between the three strata for all three conditions (*p* < 0.05). Based on genetic risk alone, we identified not only a high-risk group, but also a group at elevated risk for all health conditions. These decision rule models comprising blood biomarkers, physical measurements, and polygenic risk scores moderately improve commonly used clinical risk scores at identifying individuals likely to benefit from lifestyle intervention for three of the most common lifestyle-related chronic health conditions. Their utility as part of digital data or digital therapeutics platforms to support the implementation of lifestyle interventions in preventive and primary care should be further validated.

## 1. Introduction

Developed countries have seen a consistent rise in life expectancy and overall improving trends in chronic disease outcomes [1]. In just six decades, this has translated to a global increase in life expectancy of over 20 years for both men and women [1]. Yet, longer life expectancy has been accompanied by an increase in the prevalence of common chronic diseases, such as coronary artery disease (CAD), type 2 diabetes (T2D), and hypertension, which pose a significant burden to societies and limit healthy life expectancy (HALE) both with regards to morbidity and mortality [2,3]. Preventive strategies which allow for earlier lifestyle intervention are a solution to tackle the growing burden of lifestyle-related health conditions. Indeed, lifestyle interventions such as weight loss, limiting (saturated) fat intake, and 30 min of exercise per day are recommended across multiple guidelines to reduce cardiovascular disease risk and the progression from prediabetes to T2D [4,5]. Yet, the sustainable implementation of lifestyle interventions faces several challenges, and cannot be achieved with one-size-fits-all approaches [6]. Rather, adherence and maintenance of health behavior change requires personalized lifestyle recommendations.

To be able to provide such targeted lifestyle recommendations, the first step is to adequately stratify risk in individuals in a pre-clinical state and prioritize which aspects of their health they ought to focus on. For the three prevalent chronic health conditions mentioned above, several risk assessment tools have been made available to primary care physicians, including the Framingham risk scores [7,8,9]. These risk scores incorporate clinical and laboratory parameters, and have been shown to perform comparably well in European populations to other risk scores [7,10]. However, over two thirds of models for cardiovascular risk are restricted to a mixture of demographics, medical history, blood pressure and lipid profile, and a limited set of lifestyle factors, such as smoking [11]. Until now, these models have not included physical measurements or genetic susceptibility, although these health conditions are known to be multifactorial in nature, and for instance, progression from prediabetes to T2D is accelerated by even modest increases in adiposity, in individuals at higher genetic risk [6]. Especially when several studies have shown that the addition of genetic risk scores, as well as scores combining physical measurements and lifestyle factors, to demographic and biomarker data can improve risk stratification in preventive and primary care settings [11,12,13,14,15,16,17].

This study aimed to evaluate decision rules models incorporating other routine biomarkers, physical measurements, and genetic information in addition to established risk factors and investigate whether these improve risk stratification for three prevalent lifestyle-related health conditions in the large population-based UK Biobank cohort.

## 2. Materials and Methods

### 2.1. Study Population

The UK Biobank is a longitudinal population-based cohort of 502 503 participants aged between 37–73 years old, collected between 2006 and 2010. For this study, we included only participants without coronary artery disease, T2D, and hypertension diagnosed by a physician at recruitment, in whom extensive follow-up data were available. In addition, individuals without diagnosed disease but who at baseline crossed a “clinical threshold” for any of the health conditions were also excluded from further analysis. These were individuals with at least grade 1 hypertension (i.e., a systolic blood pressure equal to or greater than 140 mmHg and/or a diastolic pressure equal to or greater than 90 mmHg) [18], a fasting glucose value above 7.0 mmol/L for T2D [19], and individuals with significantly impaired kidney function assessed as an age-dependent function glomerular filtration (eGFR) and microalbuminuria according to European and international guidelines [20,21].

Individuals for whom any of the variables in Table 1 were missing were also excluded. This study was conducted under UK Biobank application 55,495. Local Institutional Review Board ethics approval was not necessary for this study.

### 2.2. Biomarkers, Physical Measurements and Polygenic Risk Scores

**Figure 1:**
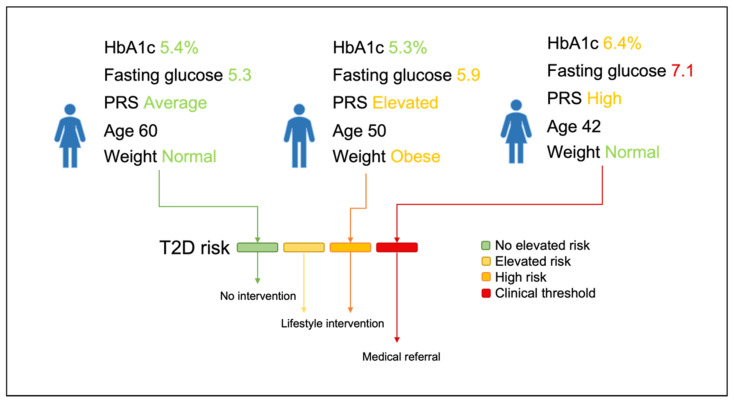
Schematic overview of how individuals are classified according to the rules models into four risk categories. Those without any (bio)markers, including genetic scores and physical measurements, are not at elevated risk; those with a specific set of literature-based risk factors as detailed in Table S2 are at high risk; others with markers outside of normal range but without decisively high risk factors, are at elevated risk. When a marker such as glucose crosses the clinical threshold, participants would no longer be recommended lifestyle intervention. HbA1c = glycated hemoglobin, PRS = polygenic risk score, T2D = diabetes type 2.

To define the risk factors for each of the health conditions, a literature search was conducted in accordance with the 2009 Preferred Reporting Items for Systematic Reviews and Meta-Analyses (PRISMA) statement [21]. We searched for meta-analyses indexed in PubMed that were published between January 2014 and October 2021 (additional details on the search strategy available in Table S1, and PRISMA flowcharts in Figures S1–S3). We also searched relevant national and international clinical guidelines not originally identified by the search. Based on the findings of the literature review, rules were defined to stratify individuals as high, elevated, and no elevated risk. An overview of the rules models is given in Figure 1, using T2D as an example.

These rules are then described in more detail in Table S2, and shortly below. Data on biomarkers were retrieved from the blood biochemistry category in the UKB, physical measurements from the body size measurements and abdominal composition categories, and smoking status was ascertained based on the self-reported smoking status registered at recruitment. Family and medical history were retrieved from the respective categories.

### 2.3. Coronary Artery Disease

For coronary artery disease, the literature and additional guideline search identified total cholesterol [22], HDL cholesterol [7,23,24], LDL cholesterol [24], triglycerides [23,25,26,27], and high-sensitivity C-reactive protein (hs-CRP) [28,29,30] as relevant blood biomarkers. The Framingham Risk Score for 10-year coronary heart disease risk was used, which included information on treatment for hypertension and smoking status [7]. A polygenic risk score for coronary artery disease was calculated as described below. Individuals were classified as high risk for which intervention is advised if they met any of the following rules, all weighted equally: total cholesterol above 8 mmol/L [20,21], systolic blood pressure above 180 mmHg [20], LDL cholesterol above 4.9 mmol/L [20,21], and if triglycerides and/or hs-CRP were out of range [26,27,28] and either the incidence risk according to Framingham or the genetic susceptibility score were “high”. The no elevated risk profile was defined as no biomarkers being out of range, the genetic susceptibility score below the eighth decile, and negative family history. All others for which at least one risk factor was elevated were classified as intermediate risk.

### 2.4. Type 2 Diabetes

Glycaemic variables (fasting glucose and HbA1c), blood lipids, markers of body composition, blood pressure, family history, gender, and smoking were identified as risk factors [8,31,32,33,34,35]. The Framingham Risk Score for diabetes was used, and a polygenic risk score for diabetes was calculated [8]. Participants were placed in the highest stratum if they met any of the following rules, all weighted equally: HbA1c was above 6.5% and fasting glucose was below 6.1 mmol/L, fasting glucose was above 6.1 mmol/L, either of the glycemic variables was elevated (HbA1c between 5.5% and 6.4% or fasting glucose between 5.6 mmol/L and 6.1 mmol/L) and they were overweight/obese, their clinical risk was high, their glucose was unregulated, and their genetic susceptibility was high, or if their clinical risk was elevated, they were older than 45, had a HDL cholesterol below 0.9 mmol/L, and triglycerides above 2.8 mmol/L [19]. Participants were classified as not being at elevated risk if all biomarkers were within normal range, the genetic susceptibility score was below the eighth decile, and clinical risk was not elevated. All others with at least one marker out of range were considered at intermediate risk.

### 2.5. Hypertension

For hypertension, the literature and additional guideline search identified age [36], systolic and diastolic blood pressure [36,37,38,39,40,41], body mass index (BMI) [42,43], gender [44], and smoking status as relevant markers. The Framingham Risk Score for hypertension risk [9] was used, and a PRS for systolic blood pressure was calculated. Participants were classified as high risk if their systolic blood pressure was high normal (130 to 139 mmHg), and/or the diastolic pressure was between 80 and 89 mmHg [41]. Equally, those with a high clinical risk, or an elevated clinical risk and a high PRS were stratified as high risk. The no elevated risk profile was defined as all biomarkers being within normal range, the genetic susceptibility score being below the eighth decile, and incidence risk according to the clinical score not being elevated. All others with at least one marker out of range were considered at intermediate risk.

### 2.6. Polygenic Risk Scores

Polygenic risk scores (PRS) were calculated using an additive model for CAD, T2D, and hypertension. Individuals were binned into deciles based on their PRS scores and the average disease incidence was calculated for each decile. The difference between individuals in the tenth risk decile, those in the nineth and eighth deciles, and all other deciles were assessed. The 1000 Genomes dataset was used as reference panel for the linkage disequilibrium (LD) calculations [45]. Linkage disequilibrium refers to the non-random association of alleles at different loci, and can cause associations between multiple alleles and a given phenotype to tag the same effect in genome-wide association studies (GWAS). When calculating a PRS, the effect of each single nucleotide polymorphisms (SNPs) is included in the calculation despite some of the SNPs tagging the same effect. By applying the LDpred tool when calculating PRS, the SNP weights/effects are corrected based on the LD, which minimizes the inflation of the estimated risk through the repeated addition of the same effect across different SNPs [46]. The genotyping data and data containing the tested phenotype outcomes were downloaded from the UKB. All variants with an imputation correlation (R^2^) below 0.4 determined with the minimac3 algorithm, were removed from the downloaded genotyping files [47]. Imputation is a technique which allows for the accurate evaluation of associations at genetic markers that are not directly genotyped, thereby increasing the power of GWAS, particularly when combining results across studies that rely on different genotyping platforms [48]. This 0.4 threshold is a commonly used threshold for the R^2^ between imputed genotype calls and the true underlying genotypes [49].

Summary statistics files from three large GWAS conducted in other cohorts were used to calculate PRS for CAD, T2D, and hypertension [50,51,52]. These publicly available summary statistics were reformatted where necessary to be consistent with the format required by LDpred. A rho of 1 was used, and all variants with a GWAS significance *p*-value below 0.01 were selected based on previous studies showing marginal differences between this and other stringency cutoffs (Table S3) [13]. In total, the T2D, CAD, and hypertension PRS included 199,120, 139,885, and 400,016 SNPs, respectively. The PRS were also computed with and without adjustment of the following variables: genotyping array, first four principal components, age and sex. To assess the added predictive value of PRS over sex and age alone, we also added the predictions of a logistic regression model including only sex and age. Individuals were binned into deciles based on their PRS scores and the average disease incidence at each age was calculated for each decile. Additional methods are available in the Supplementary Materials.

### 2.7. Ascertainment of Disease Incidence

Information regarding the variables used to calculate incidence for each of the health conditions at 8 years after study enrollment can be found in Table S4. In short, we used International Statistical Classification of Diseases and Related Health Problems (ICD) codes, and the self-reported diagnoses collected at recruitment and follow-up questionnaires.

### 2.8. Statistical Analysis

Similar to the three strata of risk for the decision rules model, three risk strata (“low”, “intermediate”, and “high”) were defined for the Framingham scores. For the coronary artery disease risk score, the bottom, middle, and top tertiles were used as risk categories [7]. For diabetes, categories were based on <3%, 3% to 8%, and >8% incidence risk at eight-years [8]. For hypertension, this was <5%, 5% to 10%, and >10% incidence risk [9].

To evaluate the ability to discriminate higher risk individuals who would be suggested lifestyle intervention from those at no elevated risk, we used the area under the receiver operating characteristics (AUROC) curve computed from 2000 bootstrap iterations. Sensitivity and specificity for each model is also presented. Additionally, the Net Reclassification Index (NRI), with the number of individuals at low risk or recommended lifestyle intervention in the initial against the updated model, and the net improvement in risk classification in individuals who developed and did not develop the disease, were calculated to evaluate potential additional predictive value of the suggested models compared to the Framingham scores [53,54].

Cox proportional hazards models were used to test the association of risk strata defined by the decision rules model and the clinical scores with incident events of CAD, T2D, and hypertension. Hazard ratios (HRs) with 95% confidence intervals were calculated between risk strata and the reference group (those not at elevated risk for the decision rules model, and low risk for Framingham).

We considered a *p*-value < 0.01 as statistically significant for differences in AUROC determined by DeLong’s nonparametric test and *p*-value < 0.05 significant for differences in risk between strata [55]. All data analyses were performed using R software v4.0.3 and the “*survival*”, “*survminer*”, “*predictABEL*”, and “*ggplot2*” packages were used to conduct the survival analysis and generate graphs [56].

## 3. Results

### 3.1. Population Characteristics

In total, 60,782 unique participants had follow-up data available, of which 42,978, 36,913, and 33,541 were included in the analyses for T2D, CAD, and hypertension, respectively. Table 1 shows the baseline characteristics of the study population and the cohort selection process is illustrated in Figure S4.

During a median follow-up of 8.8 years (5th and 95th percentile, 8.7–8.8), 500 incident CAD cases in 300,407 person-years, 1005 incident T2D cases in 347,382 person-years, and 2379 incident hypertension cases in 274,009 person-years were ascertained. Participants were aged 56.3 years on average, and slightly more participants were female (51.2%). Average values for all lipid markers were above general guidelines [21]. Similarly, physical measurements of BMI and waist circumference were above the existing thresholds for abdominal obesity, and both average systolic and diastolic blood pressure values crossed the stage 1 hypertension threshold [57,58].

### 3.2. Polygenic Risk Scores

**Figure 2:**
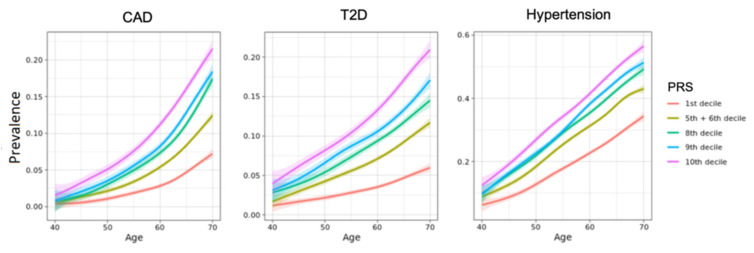
Cumulative incidence in different risk strata. Risk classification conducted based on the logistic regression model of the PRS adjusted for age, sex, first four principal components, and array type.

For all three health conditions, a higher PRS was strongly associated with a higher incidence rate (Figure 2).

For the highest risk stratum compared to the rest of the population, this translated to a hazard ratio (HR) of 4.6 (95% CI 3.8–5.6), 2.9 (2.5–3.4), and 1.9 (1.7–2.1) for CAD, T2D, and hypertension, respectively (Table 2).

When comparing the highest risk individuals to those in the first seven deciles, the HRs were 7 (95% CI 5.7–8.7), 3.8 (3.2–4.4), and 2.2 (2.0–2.5) (Table 3). The risk for individuals in the ninth and eighth deciles was also significantly higher compared to individuals in the first seven deciles, with HRs of 3.4 (2.7–4.2) for CAD, 2.3 (2.0–2.7) for T2D, and 1.8 (1.7–2) for hypertension (Table 3). These results were also seen when calculating PRS for the entire UKB cohort, without selecting sub-populations with follow-up data (Table S5).

### 3.3. Sensitivity Analysis

The Framingham scores achieved an AUROC of 0.67 (95% CI 0.63–0.71) for women and 0.60 (0.58–0.63) for men, 0.72 (0.71–0.73), and 0.60 (0.59–0.60) for CAD, T2D, and hypertension, respectively. Sensitivity and specificity for these models were 59.3% and 74.4%, 49.5% and 70.8%, 72.2% and 71.6%, and 97.4% and 22.1%. The performance of the decision rules model was better than Framingham for CAD in men, T2D, and hypertension with an AUROC of 0.66 (0.64–0.68) for CAD, 0.75 (0.74–0.76) for T2D, and 0.70 (0.69–0.71) for hypertension (Table 2, *p* < 0.01 for all). There was no difference in performance between the decision rules model and Framingham for CAD in women. The discriminatory power of the decision rules model was also superior to PRS alone (Table 3). Specificity was higher for T2D (68.2, 67.7–68.6%) and hypertension (65.5, 65–66%), with sensitivity also higher for T2D (81.5, 79.1–84%) and lower for hypertension (73.9, 72.7–75.7%). For CAD, the decision rules model achieved higher sensitivity (72.0, 68.0–76.0%) but lower specificity (59.8, 59.3–60.3%) than both Framingham models. For the decision rules models, positive predictive values were higher for T2D, hypertension and CAD in women, but lower than for the men’s model (Table S6). Negative predictive values were extremely high for all models, with the highest being 99.58% (99.50–99.65%) for the Framingham for CAD in women and the lowest 97.05% (96.85–97.24%) for the decision rules for hypertension (Table S6).

### 3.4. Risk Stratification and Lifestyle Advice Recommendations

**Figure 3:**
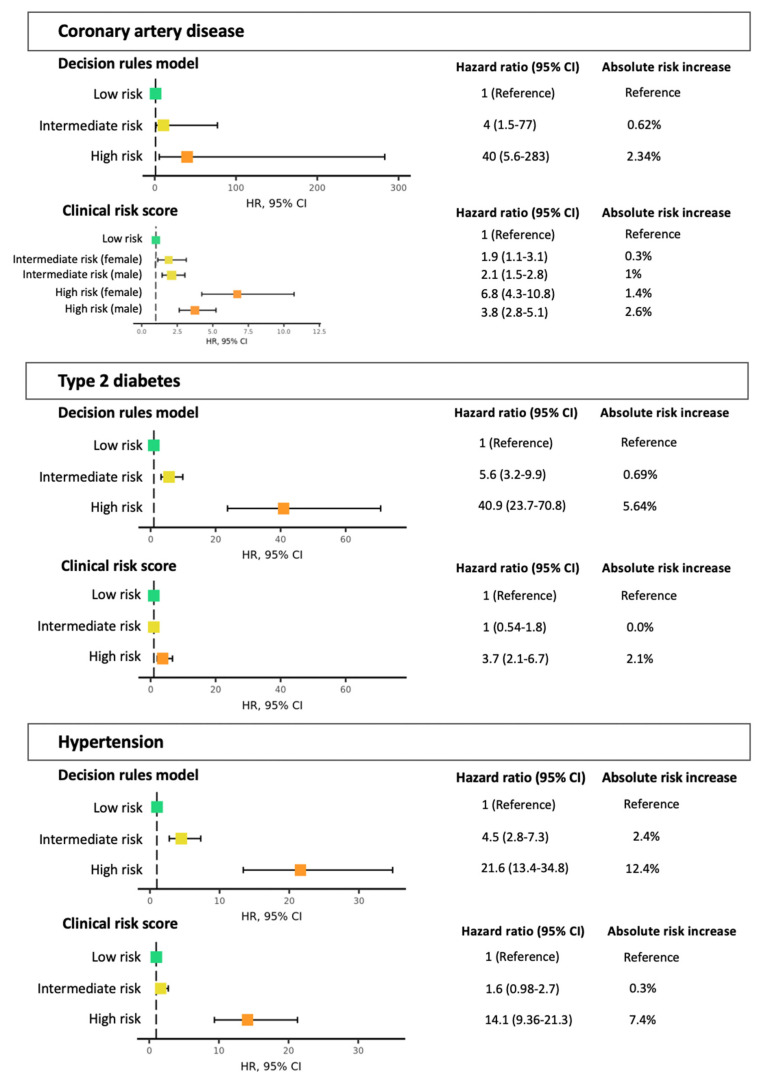
Hazard ratios (HR) of disease incidence per risk stratum. The group with no risk factors is used as reference. Both the HR and the absolute risk are displayed for each decision rules model and clinical score for all.

The observed absolute risk for each health condition differed between the high, intermediate, and low risk strata for the decision rules model, but not for the Framingham risk score (Figure 3).

In terms of absolute risk, being classified as high risk by the clinical score translated to a 2.6% and 1.4% difference in absolute risk compared to not being at elevated risk for CAD in men (HR 3.8, 2.8–5.1) and in women (HR 6.8, 4.3–10.8), 2.1% for T2D (HR 3.7, 2.1–6.7), and 7.4% for hypertension (HR 14.1, 9.36–21.3). For the intermediate risk stratum, there was a risk difference for CAD in men and women, but not for T2D or hypertension. In comparison, the high-risk group in the decision rules model showed a 2.34% increase in absolute risk for CAD (HR 40, 5.6–283), 5.64% for T2D (HR 40.9, 23.7–70.8), and 12.4% for hypertension (HR 21.6, 13.4–34.8). For the intermediate risk group, these differences were 0.62% (HR 4, 1.5–77), 0.69% (HR 5.6, 3.2–9.9), and 2.4% (HR 4.5, 2.8–7.3), respectively. If all individuals in the higher risk group were recommended lifestyle intervention as a consequence of their baseline measurements, 40.6%, 33%, and 37.2% of all individuals would be recommended lifestyle intervention for CAD, T2D, and hypertension with the decision rules model. For T2D and hypertension, this is 41.6% and 53% less than if the Framingham risk scores were used, while detecting as many cases for T2D and only 561 fewer for hypertension. This translated to a NRI of 5.8 (95% CI 2.4–9.3; *p* < 0.001), and to a NRI of 19.9 (18.1–21.8; *p* < 0.001) for hypertension (Table 4). For CAD, 14,980 (40.6%) individuals would have been recommended intervention by the decision rules, compared to 10,111 (27.4%) for Framingham. This represents a difference of detecting and advising intervention to 72% of all those who eventually developed disease, as opposed to 53.2%. In addition, 15.4% of those who ended up developing CAD were classified as low risk by Framingham, compared to 0.2% for the decision rules model. This did, however, not translate to significantly improved net reclassification, with a NRI of 5.6% (−1.4–12.7; *p* = 0.12) and 0.8% (−7.5–9.1; *p* = 0.85) for men and women, respectively (Table 4).

## 4. Discussion

We investigated the association of different risk categories of three decision rule models incorporating blood biomarkers, physical measurements, and genetic information, with incident disease for three common lifestyle-related health conditions and compared its performance to currently used clinical risk calculators in 60,782 returning participants in the population-based UK Biobank study. Individuals classified as high risk who would be recommended lifestyle intervention by the decision rules model had a 40-, 41-, and 22-fold higher 8-year risk of CAD, T2D, and hypertension compared to those who were classified as not having elevated risk. All decision rules models either outperformed the respective Framingham clinical score or showed improvement in the detection of cases likely to benefit from lifestyle intervention.

We showed that adding other biomarkers, physical measurements, and genetic risk to traditional clinical risk scores leads to a slight improvement in predictive performance for all three health conditions measured by sensitivity analysis, and to a net reclassification benefit for T2D and hypertension. From the many clinical risk scores for risk estimation of cardiometabolic health conditions available, we chose to compare our decision rules models to the Framingham risk scores due to their extensive validation across multiple cohorts [10,11]. In this sub-population of the UKB cohort, the Framingham scores performed comparably to studies in North American and Dutch populations (0.63 to 0.67), but slightly worse than reports from other studies, including lower than in the best original validation studies (0.66 to 0.83) [59,60]. For hypertension, specifically, the inferior performance of the Framingham model compared to other studies likely comes from the substantially lower number of prehypertensive individuals and mean blood pressure values in these cohorts (below 120 mmHg systolic and 75 mmHg diastolic) compared to the UKB, leading to as many as 79.3% of individuals being classified as high risk [61,62]. Compared to the Framingham scores, decision rules models detected more cases likely to benefit from lifestyle intervention for diabetes and for hypertension (5 and 20%, respectively). The slight improvement in performance of the rules model for diabetes is not surprising, as unregulated HbA1c is a risk factor for disease development in prediabetics, and specific insulin resistance phenotypes are linked to central adiposity [63,64]. Similarly, there is growing evidence for genetics playing a more central role in the diabetes burden than previously thought [65,66]. For hypertension, the addition of genetic data also likely explains the improved performance of the rules model. For CAD, despite more individuals who eventually developed disease having been classified into higher risk strata by the rules models, as shown by the 51% and 70% net correct reclassifications for CAD in women and men (Table 4), there was no significant reclassification benefit overall due to the higher number of individuals classified as high risk. However, for a low-risk, high-benefit lifestyle-based intervention the improvements seen with the rules model may be meaningful in practice, as discussed below in more depth.

While modest in magnitude, the differences in performance between the different models could have significant practical implications. Preventive health programs should consider the health risks of individuals holistically across a spectrum of mental and physical health. By improving the precision to detect those who eventually developed the disease and would be recommended intervention and minimizing the number of individuals who did not develop the disease and would have nonetheless been advised to take action, these models have the potential to increase the impact of such programs in two ways. On the one hand, it could increase their effectiveness, since the number of prevented cases if the interventions were successfully implemented would be higher. On the other hand, cardiometabolic health issues are highly prevalent. By also accurately identifying individuals less likely to benefit from a cardiometabolic health intervention in the short-term, these models can be combined with models for other physical and mental health conditions and help low risk individuals to prioritize lifestyle changes in other aspects of their health. With recent studies showing that programs as short as three to five months can trigger diabetes remission and improve cardiovascular risk factors [67,68,69], the use of these stratification mechanisms for a periodic risk assessment across varied lifestyle conditions would be a valuable tool for optimizing return on investment in personalized preventive medicine programs.

With regards to the addition of genetic risk to clinical scores, our findings support recent studies that suggested adding genetic susceptibility scores to clinical scores for CAD and T2D, as well as stroke or cardiovascular disease led to improvements in risk prediction [12,70,71]. Based on genetic risk alone, we identified a group of high risk individuals with hazard ratios of 4.6-, 2.9-, and 1.9 for CAD, T2D, and hypertension. However, we also encountered differences between the top risk decile and the ninth and eighth deciles, and between these and the rest of the population. In comparison, Khera et al. identified a similar risk increase only in the top 8% and 3.5% of individuals in the UKB, for CAD and T2D respectively, and the top 5% individuals in the Finnish cohort of Mars et al. were at 2.62-fold increased risk of CAD and 3.28-fold for T2D (Table S5, Figure S5) [12,13]. This effectively demarcates not only a “high risk”, but also an “elevated risk” group in these two deciles, compared to the “no elevated risk” group comprising the rest of the population.

One of the barriers to the implementation of risk models in preventive and primary care has been the belief that such algorithms have an actual low impact on decision-making in apparently healthy individuals, and mostly generate demand for “unnecessary” care [72,73,74]. In this study, we make two significant contributions towards overcoming this issue. First, we showed that easily interpretable decision rules models including genetic risk can better identify individuals at low risk unlikely to benefit from lifestyle interventions in the short-term than traditional clinical scores. Models based on risk factor burden are easy to interpret and communicate, and a simple metric such as the absence or presence of more than one risk factor is associated with substantial differences in lifetime risk of cardiometabolic health conditions [75]. By including genetic risk in risk factor burden calculations in an additive way, we can identify individuals at genetically elevated or high risk with normal demographic and blood risk factors. There remain substantial financial and technical challenges in conducting GWAS, and in correctly calculating and interpretating PRS, for different health conditions. In individuals for whom routinely collected medical and biomarker data clearly identify a higher risk, or for monogenic conditions, the addition of polygenic risk is unlikely to bring additional useful information. However, as the GWAS and PGS catalogues keep expanding their—for now limited—repertoire of traits and conditions, this approach could be especially meaningful for implementation in preventive care, where risk stratification targets a younger, usually healthier populations [76,77].

Second, the large sample size of the UK Biobank even after exclusion of individuals without follow-up, allows us to extrapolate the potential impact of these models for preventive lifestyle intervention at large scale. In the Netherlands, more than 16,000 people enrolled themselves in a combined lifestyle intervention program between January 2019 and April 2020 alone. In a UKB population at least twice that, 9000 and 14,000 fewer people would have been recommended lifestyle intervention by the decision rules compared to the clinical risk scores for T2D and hypertension. With a growing number of digital medical data and digital therapeutics platforms available to support clinicians and empower individuals to proactively act on their health, it is becoming easier to collected, process and analyze data from different sources such as the blood, body composition, and genetic markers evaluated in this study. When integrated with such platforms, the models developed in this study represent a viable, potentially less resource intensive framework for lifestyle interventions in preventive and primary care. A possible criticism of the inclusion of PRS and additional markers in the suggested models is that their improvement in prediction comes with an increase in complexity of the model and potentially higher costs for the individual and/or health system. While both points have received attention from the general public and scientific community, the growing literature on the challenges and solutions for the implementation of complex models including PRS in clinical practice contrasts with that on its cost-effectiveness, with only few examples in cancer screening [78,79]. On the former, different recent studies have shown the addition of PRS to conventional risk factors leads to improvements in prediction which, while modest, could translate into meaningful clinical benefit if applied at scale compared to conventional risk factors alone [80], and that PRS alone or PRS combined with age and sex across different ages performs comparably to established clinical scores such as the pooled cohort equations model [81]. As such, we are not suggesting these models replace screening methods which are effective, inexpensive, and safe [82]. Instead, we see the possible applications of these models within two contexts. In the shorter term, as an additional offer to individuals in the booming consumer health market mentioned above which has seen widespread interest in health programs based on genetics and biological and digital health data [83,84,85]. And later, as the costs for genetic analysis keep decreasing and use of genetic information in primary and secondary care settings grows, for example, through the adoption of pharmacogenetic passports [86,87], targeted prevention strategies for those in possession of their genetic data from an earlier age or in settings of unavailability or low uptake of screening programs could become cost-effective [81].

This study also presented some limitations. Firstly, the list of risk factors included is not exhaustive, due to both the high level of evidence required for inclusion in the model (most studies considered were meta-analyses) as well as the non-availability of other relevant variables in the UK Biobank data repository. Secondly, there is an inherent risk of bias to this study, due to the selection of participants for whom voluntarily provided follow-up data was available, as well as the known selection bias to biobank studies [88]. Thirdly, being a decision rules model, our proposed model does not provide individual risk predictions. While this increases the interpretability and applicability of the model (especially in a primary and preventive care setting), individuals within the same stratum may have different actual risk. Fourthly, we conducted the analysis with the assumption that all individuals classified as high risk who would have been recommended lifestyle intervention would not only have started it, but also achieved some degree of success. With a growing offer of consumer health and wellbeing programs, as well as employer-sponsored health programs, it is easier than ever before for individuals to preventively implement lifestyle changes [89]. However, many factors not accounted for here play a role in determining the actual effectiveness of these programs, so prospective validation in a study setting as well as in the market is required to assess the actual impact of these models on the effectiveness of preventive health interventions. Fifth, both the GWAS for the three PRS used in this study, as well as the UK Biobank cohort itself, are very ethnically homogeneous, with more than 90% of total participants being of white ethnicity and European descent. Therefore, the PRS results for UK Biobank participants of other ethnicities may be sub-optimal, and PRS and model validation will be required in cohorts with more diverse ethnical background. Lastly, this study had limited information on prevention strategies or therapies undergone by study participants during follow-up. Consequently, participants’ risk for any of the outcomes may have been modulated by medication and lifestyle or other interventions which are not accounted for in the analysis. This possible bias is especially present for CAD since pharmacological interventions do not form part of standard preventive strategies for hypertension or diabetes, but statin prescription is a staple of cardiovascular risk management in individuals with elevated to high LDL cholesterol who would be classified as high risk by the model. To further investigate this, we analyzed medication prescriptions for all three conditions during follow-up in individuals in the low and high risk groups who had not been diagnosed with the condition (i.e., who reported medication not present at baseline but present at follow-up without an accompanying diagnosis). This analysis showed no difference in medication usage between these two risk strata for T2D and that only a small fraction of individuals classified as low risk as baseline (1% and 0.3% for hypertension and CAD) started medication during follow-up. This was significantly less than those in the high risk group (2.6% and 6.3%, respectively), indicating these limitations are unlikely to have meaningfully affected the reported results.

In conclusion, in this prospective population-based cohort study of 60,782 people, we developed and validated three risk stratification models for three prevalent chronic conditions. Adding other blood markers, physical measurements, and genetic susceptibility scores to currently used clinical risk scoring tools resulted in moderate improvements in performance and in the identification of individuals likely to benefit from lifestyle intervention. When integrated with digital data or digital therapeutics platforms that enable the collection and analysis of these data, these algorithms can be used to support the successful adoption of lifestyle interventions in preventive and primary care.

## Figures and Tables

**Table 1:** Baseline characteristics table.

Characteristic	Mean (SD), Percentage (%), or Number of Participants (N)
Total, No.	60,782
Age, y	56.3 (7.59)
Female	51.2%
With CAD at follow-up	*n* = 500
With diabetes at follow-up	*n* = 1005
With hypertension at follow-up	*n* = 2379
Blood pressure, mm Hg	
Systolic	138 (19)
Diastolic	82 (10)
Smoking	
Ideal (never or stopped >1 y ago)	82.7%
Intermediate (stopped <1 y ago)	0.3%
Current smoker	5.6%
Body composition	
BMI	26.8 (4.35)
Waist circumference (cm)	88.7 (12.8)
Waist-to-hip ratio	0.86 (0.09)
Body fat percentage (%)	30.2 (8.3)
Blood biomarkers	
Total cholesterol (mmol/L)	5.71 (1.1)
LDL cholesterol (mmol/L)	3.58 (0.84)
HDL cholesterol (mmol/L)	1.47 (0.38)
Triglycerides (mmol/L)	1.68 (0.97)
hs-CRP (mg/L)	2.17 (3.8)
Fasting glucose (mmol/L)	5.0 (1.0)
HbA1c (mmol/mmol)	35.2 (5.3)
Albumin-creatinine ratio	2.4 (8.3)
Family history	
No family history of diabetes	83.2%
Family history of diabetes (1 parent)	16.8%
Family history of diabetes (both)	0.01%
No family history of CAD	58.1%
Family history of CAD (1 parent)	41.8%
Family history of CAD (both)	0.07%
No family history of hypertension	57.3%
Family history of hypertension (1 parent)	42.7%
Family history of hypertension (both)	0.08%

Continuous variables are reported as mean and standard deviation (SD), and categorical variables as %. Abbreviations: CAD = coronary artery disease, BMI = body mass index, hs-CRP = high-sensitivity C-reactive protein, HbA1c = glycated hemoglobin.

**Table 2:** Risk increase for the individuals at high genetic risk (10th decile), compared to individuals at low genetic risk (1–7th decile) of population. Second and third column show hazard ratios calculated based on a logistic regression model adjusted for the respective variables. In all cases the difference with the remainder of the population was statistically significant (*p*-value < 0.01).

Health Condition	Unadjusted PRS	PRS Adjusted for 4 PCs and Array Type	PRS Adjusted for 4 PCs, Array Type, Sex and Age	Age and Sex
CAD	1.66 (0.93–2.35)	2.25 (1.39–3.11)	4.43 (3.14–5.74)	2.55 (1.63–3.47)
T2D	1.86 (1.33–2.39)	2.61 (1.96–3.26)	2.81 (2.12–3.50)	1.47 (1.00–1.94)
HT	1.37 (1.06–1.62)	1.61 (1.30–1.92)	1.77 (1.45–2.09)	1.50 (1.21–1.79)

Abbreviations: PRS = polygenic risk scores, PC = genetic principal component, CAD = coronary artery disease, T2D = type 2 diabetes, HT = hypertension.

**Table 3:** Area under the receiver operating characteristic (AUROC) curve for model discrimination of higher risk individuals amenable to lifestyle intervention assessed for the clinical risk score(s), PRS, and decision rules model. Number of individuals classified as low and higher risk and number of individuals who had developed disease at follow-up are also presented.

Model/Health Condition	Low Risk (N)	Low Risk Who Developed Disease (N)	Advised Lifestyle (N)	Advised Lifestyle Who Developed Disease (N)	AUROC (95% CI)
CAD (*n* = 21,969 women, 167 cases; 14,944 men, 333 cases)					
FRS women	7426	22	5680	99	0.67 (0.63–0.71)
FRS men	5171	55	4431	165	0.60 (0.58–0.63)
PRS	25,839	173	3692	165	0.62 (0.60–0.64)
Rule model	1521	0 ^$^	14,980	360	0.66 (0.64–0.68)
Diabetes (*n* = 42,978, 1005 cases)					
FRS	12,305	39	12,634	726	0.72 (0.71–0.73)
PRS	30,084	467	4298	239	0.57 (0.56–0.58)
Rule model	8351	13	14,169	819	0.75 (0.74–0.76)
Hypertension (*n* = 33,541, 2379 cases)					
FRS	3359	23	26,587	2317	0.60 (0.59–0.60)
PRS	23,479	1327	3354	391	0.54 (0.53–0.54)
Rule model	2274	17	12,506	1759	0.70 (0.69–0.71)

Abbreviations: *n* = number; FRS = Framingham risk score; PRS = polygenic risk score. AUROC reported with 95% confidence interval. ^$^ computed as 1 for statistical analysis purposes.

**Table 4:** Reclassification table for the decision rules models against Framingham risk scores. Number of individuals moving to new strata based on the updated models, split by events and non-events.

Model/ Health Condition	Event			Non-Event		
CAD women (*n* = 21,969,167 cases)						
	Rules model			Rules model		
Framingham	Rec. intervention	Low-risk	Corr. reclass. (%)	Rec. intervention	Low-risk	Corr. reclass. (%)
Rec. intervention	82	17	17%	3746	1835	33%
Low risk	35	33	51%	4010	12,211	25%
CAD men (*n* = 14,944,333 cases)						
	Rules model			Rules model		
Framingham	Rec. intervention	Low-risk	Corr. reclass. (%)	Rec. intervention	Low-risk	Corr. reclass. (%)
Rec. intervention	125	40	24%	2558	1708	40%
Low-risk	118	50	70%	4306	6039	42%
T2D (*n* = 42,978, 1005 cases)						
	Rules model			Rules model		
Framingham	Rec. intervention	Low-risk	Corr. reclass. (%)	Rec. intervention	Low-risk	Corr. reclass. (%)
Rec. intervention	617	109	15%	6183	5725	48%
Low-risk	202	77	72%	7167	22,898	24%
Hypertension (*n* = 33,541, 2379 cases)						
	Rules model			Rules model		
Framingham	Rec. intervention	Low-risk	Corr. reclass. (%)	Rec. intervention	Low-risk	Corr. reclass. (%)
Rec. intervention	1751	566	24%	10,477	13,793	57%
Low-risk	8	54	13%	270	6622	4%

Abbreviations: CAD = coronary artery disease, Rec. intervention = number of individuals who would have been recommended lifestyle intervention; Corr. reclass. = % of cases correctly reclassified, T2D = type 2 diabetes.

## Data Availability

The data that support the findings of this study are available from the UK Biobank project site, subject to registration and application process. Further details can be found at https://www.ukbiobank.ac.uk.

## Supplementary Materials

The following are available online at https://www.mdpi.com/article/10.3390/jpm11121322/s1, Table S1: Search strategy and results, Figure S1: PRISMA flow for the search for coronary artery disease, Figure S2: PRISMA flow for the search for hypertension, Figure S3: PRISMA flow for the search for diabetes, Table S2: Literature-based risk strata for each health condition, Supplementary Methods 1: Polygenic risk score calculation, Table S3: Summary statistics data, Table S4: UKB columns used to calculate disease incidence, Figure S4: Selection process patient population for the analyses for different health conditions, Table S5: Risk for the individuals in the highest risk decile for each genetic risk score, compared to rest of population for the complete UKB population (*n* = 442 687), Table S6: Sensitivity analysis for all models. Sensitivity, specificity, positive predictive value (PPV), and negative predictive (NPV) value all reported with 95% confidence interval, Figure S5: Prevalence of disease of individuals classed into different risked strata based on their genetic susceptibility scores.

## References

[1] GBD 2017 Mortality Collaborators (2018). Global, regional, and national age-specific mortality and life expectancy, 1950–2017: A systematic analysis for the Global Burden of Disease Study 2017. Lancet.

[2] WHO Health Systems Performance Assessment: Debates, Methods and Empiricism. https://www.who.int/publications/2003/hspa/en/.

[3] James S.L., Abate D., Abate K.H., Abay S.M., Abbafati C., Abbasi N., Abbastabar H., Abd-Allah F., Abdela J., Abdelalim A. (2018). Global Burden of Disease Global, regional, and national incidence, prevalence, and years lived with disability for 354 diseases and injuries for 195 countries and territories, 1990–2017: A systematic analysis for the Global Burden of Disease Study 2017. Lancet.

[4] Piepoli M.F., Hoes A.W., Agewall S., Albus C., Brotons C., Catapano A.L., Cooney M.-T., Corrà U., Cosyns B., Deaton C. (2016). 2016 European Guidelines on cardiovascular disease prevention in clinical practice: The Sixth Joint Task Force of the European Society of Cardiology and Other Societies on Cardiovascular Disease Prevention in Clinical Practice (constituted by representatives of 10 societies and by invited experts) Developed with the special contribution of the European Association for Cardiovascular Prevention & Rehabilitation (EACPR). Eur. Heart J..

[5] Franklin B.A., Cushman M. (2011). Recent Advances in Preventive Cardiology and Lifestyle Medicine. Circulation.

[6] Magkos F., Hjorth M.F., Astrup A. (2020). Diet and exercise in the prevention and treatment of type 2 diabetes mellitus. Nat. Rev. Endocrinol..

[7] Wilson P.W., D’Agostino R.B., Levy D., Belanger A.M., Silbershatz H., Kannel W.B. (1998). Prediction of coronary heart disease using risk factor categories. Circulation.

[8] Wilson P.W., Meigs J.B., Sullivan L., Fox C.S., Nathan D.M., D’Agostino R.B. (2007). Prediction of incident diabetes mellitus in middle-aged adults: The Framingham Offspring Study. Arch. Intern. Med..

[9] Parikh N.I., Pencina M.J., Wang T.J., Benjamin E.J., Lanier K.J., Levy D., D’Agostino R.B., Kannel W.B., Vasan R.S. (2008). A risk score for predicting near-term incidence of hypertension: The Framingham Heart Study. Ann. Intern. Med..

[10] Damen J.A., Pajouheshnia R., Heus P., Moons K.G.M., Reitsma J.B., Scholten R.J.P.M., Hooft L., Debray T.P.A. (2019). Performance of the Framingham risk models and pooled cohort equations for predicting 10-year risk of cardiovascular disease: A systematic review and meta-analysis. BMC Med..

[11] Damen J.A., Hooft L., Schuit E., Debray T.P.A., Collins G.S., Tzoulaki I., Lassale C.M., Siontis G.C.M., Chiocchia V., Roberts C. (2016). Prediction models for cardiovascular disease risk in the general population: Systematic review. BMJ.

[12] Mars N.J., Koskela J.T., Ripatti P., Kiiskinen T.T.J., Havulinna A.S., Lindbohm J.V., Ahola-Olli A., Kurki M., Karjalainen J., Palta P. (2020). Polygenic and clinical risk scores and their impact on age at onset of cardiometabolic diseases and common cancers. Nat. Med..

[13] Khera A.V., Chaffin M., Aragam K.G., Haas M.E., Roselli C., Choi S.H., Natarajan P., Lander E.S., Lubitz S.A., Ellinor P.T. (2018). Genome-wide polygenic scores for common diseases identify individuals with risk equivalent to monogenic mutations. Nat. Genet..

[14] Chatterjee N., Shi J., Garcia-Closas M. (2016). Developing and evaluating polygenic risk prediction models for stratified disease prevention. Nat. Rev. Genet..

[15] Rutten-Jacobs L.C., Larsson S.C., Malik R., Rannikmäe K., Sudlow C.L., Dichgans M., Markus H.S., Traylor M., MEGASTROKE Consortium, International Stroke Genetics Consortium (2018). Genetic risk, incident stroke, and the benefits of adhering to a healthy lifestyle: Cohort study of 306 473 UK Biobank participants. BMJ.

[16] Said M.A., Verweij N., van der Harst P. (2018). Associations of Combined Genetic and Lifestyle Risks with Incident Cardiovascular Disease and Diabetes in the UK Biobank Study. JAMA Cardiol..

[17] Lewis C.M., Vassos E. (2020). Polygenic risk scores: From research tools to clinical instruments. Genome Med..

[18] Whelton P.K., Carey R.M., Aronow W.S., Casey D.E., Collins K.J., Himmelfarb C.D., Sondra M., DePalma S.M., Gidding S., Jamerson K.A. (2018). 2017 ACC/AHA/AAPA/ABC/ACPM/AGS/APhA/ASH/ASPC/NMA/PCNA Guideline for the Prevention, Detection, Evaluation, and Management of High Blood Pressure in Adults: A Report of the American College of Cardiology/American Heart Association Task Force on Clinical Practice Guidelines. Hypertension.

[19] Nederlands Huisartsen Genootschap (2018). Diabetes Mellitus Type 2, Derde Herziening. https://richtlijnen.nhg.org/standaarden/diabetes-mellitus-type-2#volledige-tekst-literatuur.

[20] Grundy S.M., Stone N.J., Bailey A.L., Beam C., Birtcher K.K., Blumenthal R.S., Braun L.T., de Ferranti S., Faiella-Tommasino J., Forman D.E. (2019). 2018 AHA/ACC/AACVPR/AAPA/ABC/ACPM/ADA/AGS/APhA/ASPC/NLA/PCNA Guideline on the management of blood cholesterol: A report of the American College of Cardiology/American Heart Association Task Force on Clinical Practice Guidelines. J. Am. Coll. Cardiol..

[21] Mach F., Baigent C., Catapano A.L., Koskinas K.C., Casula M., Badimon L., Chapman M.J., De Backer G.G., Delgado V., Ference B.A. (2020). 2019 ESC/EAS Guidelines for the management of dyslipidaemias: Lipid modification to reduce cardiovascular risk. Eur. Heart J..

[22] Moher D., Liberati A., Tetzlaff J., Altman D.G., PRISMA Group (2009). Preferred reporting items for systematic reviews and meta-analyses: The PRISMA statement. Ann. Intern. Med..

[23] Di Angelantonio E., Sarwar N., Perry P., Kaptoge S., Ray K.K., Thompson A., Wood A.M., Lewington S., Sattar N., Emerging Risk Factors Collaboration (2009). Major lipids, apolipoproteins, and risk of vascular disease. JAMA.

[24] Madsen C.M., Varbo A., Nordestgaard B.G. (2017). Extreme High High-Density Lipoprotein Cholesterol Is Paradoxically Associated With High Mortality in Men and Women: Two Prospective Cohort Studies. Eur. Heart J..

[25] Third Report of the National Cholesterol Education Program (NCEP) (2002). Expert Panel on Detection, Evaluation, and Treatment of High Blood Cholesterol in Adults (Adult Treatment Panel III) Final Report. Circulation.

[26] Nordestgaard B.G., Varbo A. (2014). Triglycerides and cardiovascular disease. Lancet.

[27] Sarwar N., Danesh J., Eiriksdottir G., Sigurdsson G., Wareham N., Bingham S., Boekholdt M., Khaw K.-T., Gudnason V. (2007). Triglycerides and the Risk of Coronary Heart Disease: 10 158 Incident Cases among 262,525 Participants in 29 Western Prospective Studies. Circulation.

[28] Ikezaki H., Fisher V.A., Lim E., Ai M., Liu C.T., Adrienne Cupples L., Nakajima K., Asztalos B.F., Furusyo N., Schaefer E.J. (2019). Direct Versus Calculated LDL Cholesterol and C-Reactive Protein in Cardiovascular Disease Risk Assessment in the Framingham Offspring Study. Clin. Chem..

[29] Penson P.E., Long D.L., Howard G., Toth P.P., Muntner P., Howard V.J., Safford M.M., Jones S.R., Martin S.S., Mazidi M. (2018). Associations between very low concentrations of low-density lipoprotein cholesterol, high sensitivity C-reactive protein, and health outcomes in the Reasons for Geographical and Racial Differences in Stroke (REGARDS) study. Eur. Heart J..

[30] Arnett D.K., Blumenthal R.S., Albert M.A., Buroker A.B., Goldberger Z.D., Hahn E.J., Himmelfarb C.D., Khera A., Lloyd-Jones D., McEvoy J.W. (2019). 2019 ACC/AHA Guideline on the Primary Prevention of Cardiovascular Disease: A Report of the American College of Cardiology/American Heart Association Task Force on Clinical Practice Guidelines. Circulation.

[31] Richter B., Hemmingsen B., Metzendorf M.-I., Takwoingi Y. (2018). Development of type 2 diabetes mellitus in people with intermediate hyperglycaemia. Cochrane Database Syst. Rev..

[32] Ren Y., Luo X., Wang C., Yin L., Pang C., Feng T. (2016). Prevalence of hypertriglyceridemic waist and association with risk of type 2 diabetes mellitus: A meta-analysis. Diabetes Metab. Res. Rev..

[33] Riaz H., Khan M.S., Siddiqi T.J., Usman M.S., Shah N., Goyal A., Khan S.S., Mookadam F., Krasuski R.A., Ahmed H. (2018). Association Between Obesity and Cardiovascular Outcomes: A Systematic Review and Meta-analysis of Mendelian Randomization Studies. JAMA Netw. Open.

[34] Hashimoto Y., Hamaguchi M., Tanaka M., Obora A., Kojima T., Fukui M. (2018). Metabolically healthy obesity without fatty liver and risk of incident type 2 diabetes: A meta-analysis of prospective cohort studies. Obes. Res. Clin. Pract..

[35] Emdin C.A., Anderson S.G., Woodward M., Rahimi K. (2015). Usual Blood Pressure and Risk of New-Onset Diabetes: Evidence from 4.1 Million Adults and a Meta-Analysis of Prospective Studies. J. Am. Coll. Cardiol..

[36] Reboussin D.M., Allen N.B., Griswold M.E., Guallar E., Hong Y., Lackland D.T., MillerIII E.R., Polonsky T., Thompson-Paul A.M., Vupputuri S. (2018). Systematic Review for the 2017 ACC/AHA/AAPA/ABC/ACPM/AGS/APhA/ASH/ASPC/NMA/PCNA Guideline for the Prevention, Detection, Evaluation, and Management of High Blood Pressure in Adults: A Report of the American College of Cardiology/American Heart Association Task Force on Clinical Practice Guidelines. Circulation.

[37] Brunström M., Carlberg B. (2018). Association of Blood Pressure Lowering With Mortality and Cardiovascular Disease across Blood Pressure Levels: A Systematic Review and Meta-analysis. JAMA Intern. Med..

[38] Sundström J., Arima H., Jackson R., Turnbull F., Rahimi K., Chalmers J., Woodward M., Neal B., Blood Pressure Lowering Treatment Trialists’ Collaboration (2015). Effects of blood pressure reduction in mild hypertension: A systematic review and meta-analysis. Ann. Intern. Med..

[39] Thomopoulos C., Parati G., Zanchetti A. (2014). Effects of blood pressure lowering on outcome incidence in hypertension: 2. Effects at different baseline and achieved blood pressure levels—Overview and meta-analyses of randomized trials. J. Hypertens..

[40] Hong Z., Wu T., Zhou S., Huang B., Wang J., Jin D., Geng D. (2018). Effects of anti-hypertensive treatment on major cardiovascular events in populations within prehypertensive levels: A systematic review and meta-analysis. J. Hum. Hypertens..

[41] Williams B., Mancia G., Spiering W., Rosei E.A., Azizi M., Burnier M., Clement D.L., Coca A., de Simone G., Anna Dominiczak A. (2018). 2018 ESC/ESH Guidelines for the management of arterial hypertension: The Task Force for the management of arterial hypertension of the European Society of Cardiology (ESC) and the European Society of Hypertension (ESH). Eur. Heart J..

[42] Deng G., Yin L., Liu W., Liu X., Xiang Q., Qian Z., Xiang Q., Qian Z., Ma J., Chen H. (2018). Associations of anthropometric adiposity indexes with hypertension risk: A systematic review and meta-analysis including PURE-China. Medicine.

[43] Zho Q., Shi Y., Li Y.-Q., Ping Z., Wang C., Liu X., Lu J., Mao Z.X., Zhao J., Yin L. (2018). Body mass index, abdominal fatness, and hypertension incidence: A dose-response meta-analysis of prospective studies. J. Hum. Hypertens..

[44] Wei Y.-C., George N.I., Chang C.-W., Hicks K.A. (2017). Assessing Sex Differences in the Risk of Cardiovascular Disease and Mortality per Increment in Systolic Blood Pressure: A Systematic Review and Meta-Analysis of Follow-Up Studies in the United States. PLoS ONE.

[45] Auton A., Brooks L.D., Durbin R.M., Garrison E.P., Kang H.M., Korbel J.O., Marchini J.L., McCarthy S., McVean G.A., 1000 Genomes Project Consortium (2015). A global reference for human genetic variation. Nature.

[46] Vilhjálmsson B.J., Yang J., Finucane H.K., Gusev A., Lindström S., Ripke S., Genovese G., Loh P.R., Bhatia G., Do R. (2015). Modeling Linkage Disequilibrium Increases Accuracy of Polygenic Risk Scores. Am. J. Hum. Genet..

[47] Das S., Forer L., Schönherr S., Sidore C., Locke A.E., Kwong A., Vrieze S.I., Chew E.Y., Levy S., McGue M. (2016). Next-generation genotype imputation service and methods. Nat. Genet..

[48] Li Y., Willer C., Sanna S., Abecasis G. (2009). Genotype imputation. Annu. Rev. Genomics Hum. Genet..

[49] Purcell S., Neale B., Todd-Brown K., Thomas L., Ferreira M.A.R., Bender D., Maller J., Sklar P., de Bakker P.I., Daly M.J. (2007). PLINK: A Tool Set for Whole-Genome Association and Population-Based Linkage Analyses. Am. J. Hum. Genet..

[50] Hoffmann T.J., Ehret G.B., Nandakumar P., Ranatunga D., Schaefer C., Kwok P.Y., Iribarren C., Chakravarti A., Risch N. (2017). Genome-wide association analyses using electronic health records identify new loci influencing blood pressure variation. Nat. Genet..

[51] Nikpay M., Goel A., Won H.H., Hall L.M., Willenborg C., Kanoni S., Saleheen D., Kyriakou T., Nelson C.P., Hopewell J.C. (2015). A comprehensive 1000 Genomes-based genome-wide association meta-analysis of coronary artery disease. Nat. Genet..

[52] Scott R.A., Scott L.J., Mägi R., Marullo L., Gaulton K.J., Kaakinen M., Pervjakova N., Pers T.H., Johnson A.D., Eicher J.D. (2017). An Expanded Genome-Wide Association Study of Type 2 Diabetes in Europeans. Diabetes.

[53] Pencina M.J., Demler O.V. (2012). Novel metrics for evaluating improvement in discrimination: Net reclassification and integrated discrimination improvement for normal variables and nested models. Stat. Med..

[54] Pencina M.J., D’Agostino Sr R.B., D’Agostino R.B., Vasan R.S. (2008). Evaluating the added predictive ability of a new marker: From area under the ROC curve to reclassification and beyond. Stat. Med..

[55] DeLong E.R., DeLong D.M., Clarke-Pearson D.L. (1988). Comparing the areas under two or more correlated receiver operating characteristic curves: A nonparametric approach. Biometrics.

[56] Kundu S., Aulchenko Y.S., van Duijn C.M., Janssens A.C. (2011). PredictABEL: An R package for the assessment of risk prediction models. Eur. J. Epidemiol..

[57] Lear S.A., James P.T., Ko G.T., Kumanyika S. (2010). Appropriateness of waist circumference and waist-to-hip ratio cutoffs for different ethnic groups. Eur. J. Clin. Nutr..

[58] Flack J.M., Adekola B. (2020). Blood pressure and the new ACC/AHA hypertension guidelines. Trends Cardiovasc. Med..

[59] D’Agostino R.B., Grundy S., Sullivan L.M., Wilson P., CHD Risk Prediction Group (2001). Validation of the Framingham Coronary Heart Disease Prediction Scores. Results of a Multiple Ethnic Groups Investigation. JAMA.

[60] Koller M.T., Leening M.J.G., Wolbers M., Steyerberg E.W., Hunink M., Schoop R., Hofman A., Bucher H.C., Psaty B.M., Lloyd-Jones D.M. (2012). Development and Validation of a Coronary Risk Prediction Model for Older U.S. and European Persons in the Cardiovascular Health Study and the Rotterdam Study. Ann. Intern. Med..

[61] Kivimäki M., Batty G.D., Singh-Manoux A., Ferrie J.E., Tabak A.G., Jokela M., Marmot M.G., Smith G.D., Shipley M.J. (2009). Validating the Framingham Hypertension Risk Score: Results from the Whitehall II Study. Hypertension.

[62] Syllos D.H., Calsavara V.F., Bensenor I.M., Lotufo P.A. (2020). Validating the Framingham Hypertension Rsk Score: A 4-year follow-up from the Brazilian Longitudinal Study of the Adult Health (ELSA-Brasil). J. Clin. Hypertens..

[63] Zhang M., Zhang H., Wang C., Ren Y., Wang B., Zhang L., Yang X., Zhao Y., Han C., Pang C. (2016). Development and Validation of a Risk-Score Model for Type 2 Diabetes: A Cohort Study of a Rural Adult Chinese Population. PLoS ONE.

[64] Carroll S.J., Paquet C., Howard N.J., Adams R.J., Taylor A.W., Daniel M. (2014). Validation of continuous clinical indices of cardiometabolic risk in a cohort of Australian adults. BMC Cardiovasc. Disord..

[65] Udler M.S., Kim J., von Grotthuss M., Bonàs-Guarch S., Cole J.B., Chiou J., Anderson C.D., Boehnke M., Laakso M., Atzmon G. (2018). Type 2 diabetes genetic loci informed by multi-trait associations point to disease mechanisms and subtypes: A soft clustering analysis. PLoS Med..

[66] Ahlqvist E., Storm P., Karajamaki A., Martinell M., Dorkhan M., Carlsson A., Vikman P., Prasad R.B., Mansour Aly D., Almgren P. (2018). Novel subgroups of adult-onset diabetes and their association with outcomes: A data-driven cluster analysis of six variables. Lancet Diabetes Endocrinol..

[67] Lean M.E., Leslie W.S., Barnes A.C., Brosnahan N., Thom G., McCombie L., Peters C., Zhyzhneuskaya S., Al-Mrabeh A., Hollingsworth K.G. (2018). Primary care-led weight management for remission of type 2 diabetes (DiRECT): An open-label, cluster-randomised trial. Lancet.

[68] Kim S.E., Castro Sweet C.M., Cho E., Tsai J., Cousineau M.R. (2019). Evaluation of a Digital Diabetes Prevention Program Adapted for Low-Income Patients, 2016–2018. Prev. Chronic Dis..

[69] Athinarayanan S.J., Adams R.N., Hallberg S.J., McKenzie A.L., Bhanpuri N.H., Campbell W.W., Volek J.S., Phinney S.D., McCarter J.P. (2019). Long-Term Effects of a Novel Continuous Remote Care Intervention Including Nutritional Ketosis for the Management of Type 2 Diabetes: A 2-Year Non-randomized Clinical Trial. Front. Endocrinol..

[70] McCarthy M.I. (2017). Painting a new picture of personalised medicine for diabetes. Diabetologia.

[71] Levin M.G., Rader D.J. (2020). Polygenic Risk Scores and Coronary Artery Disease: Ready for Prime Time?. Circulation.

[72] Müller-Riemenschneider F., Holmberg C., Rieckmann N., Kliems H., Rufer V., Müller-Nordhorn J., Willich S.N. (2010). Barriers to Routine Risk-Score Use for Healthy Primary Care Patients: Survey and Qualitative Study. Arch. Intern. Med..

[73] Kappen T.H., Van Loon K., Kappen M.A., van Wolfswinkel L., Vergouwe Y., van Klei W.A., Moons K.G., Kalkman C.J. (2016). Barriers and facilitators perceived by physicians when using prediction models in practice. J. Clin. Epidemiol..

[74] Rosselo X., Dorresteijn J.A.N., Janssen A., Lambrinou E., Scherrenberg M., Bonnefoy-Cudraz E., Cobain M., Piepoli M.F., Visseren F.L., Dendale P. (2019). Risk prediction tools in cardiovascular disease prevention. Eur. J. Prev. Cardiol..

[75] Lloyd-Jones D.M., Leip E.P., Larson M.G., D’Agostino R.B., Beiser A., Wilson P.W.F., Wolf P.A., Levy D. (2006). Prediction of Lifetime Risk for Cardiovascular Disease by Risk Factor Burden at 50 Years of Age. Circulation.

[76] MacArthur J., Bowler E., Cerezo M., Gil L., Hall P., Hastings E., Junkins H., McMahon A., Milano A., Morales J. (2017). The new NHGRI-EBI Catalog of published genome-wide association studies (GWAS Catalog). Nucleic Acids Res..

[77] Lambert S.A., Gil L., Jupp S., Ritchie S.C., Xu Y., Buniello A., McMahon A., Abraham G., Chapman M., Parkinson H. (2021). The Polygenic Score Catalog as an open database for reproducibility and systematic evaluation. Nat. Genet..

[78] Liu Q., Davis J., Han X., Mackey D.A., MacGregor S., Craig J.E., Si L., Hewitt A.W. (2021). Cost-effectiveness of polygenic risk profiling for primary open-angle glaucoma in the United Kingdom and Australia. medRxiv.

[79] Wong J., Chai J.H., Yeoh Y.S., Mohamed Riza N.K., Liu J., Teo Y.-Y. (2021). Cost effectiveness analysis of a polygenic risk tailored breast cancer screening programme in Singapore. BMC Health Serv. Res..

[80] Sun L., Pennells L., Kaptoge S., Nelson C.P., Ritchie S.C., Abraham G., Arnold M., Bell S., Bolton T., Burgess S. (2021). Polygenic risk scores in cardiovascular risk prediction: A cohort study and modelling analyses. PLoS Med..

[81] Elliott J., Bodinier B., Bond T.A., Chadeau-Hyam M., Evangelou E., Moons K.G.M., Dehghan A., Muller D.C., Elliott P., Tzoulaki I. (2020). Predictive Accuracy of a Polygenic Risk Score–Enhanced Prediction Model vs. a Clinical Risk Score for Coronary Artery Disease. JAMA.

[82] Sud A., Turnbull C., Houlston R. (2021). Will polygenic risk scores for cancer ever be clinically useful?. NPJ Precis. Oncol..

[83] Huckvale K., Jason Wang C., Majeed A., Car J. (2019). Digital health at fifteen: More human (more needed). BMC Med..

[84] Gordon W.J., Landman A., Zhang H., Bates D.W. (2020). Beyond validation: Getting health apps into clinical practice. NPJ Digit. Med..

[85] Natanson E. Healthcare Apps: A Boon, Today and Tomorrow. Forbes. https://www.forbes.com/sites/eladnatanson/2020/07/21/healthcare-apps-a-boon-today-and-tomorrow/?sh=59bfd4ab1bb9.

[86] Natarajan P., Young R., Stitziel N.O., Padmanabhan S., Baber U., Mehran R., Sartori S., Fuster V., Reilly D.F., Butterworth A. (2017). Polygenic risk score identifies subgroup with higher burden of atherosclerosis and greater relative benefit from statin therapy in the primary prevention setting. Circulation.

[87] Mega J.L., Stitziel N.O., Smith J.G., Chasman D.I., Caulfield M., Devlin J.J., Nordio F., Hyde C., Cannon C.P., Sacks F. (2015). Genetic risk, coronary heart disease events, and the clinical benefit of statin therapy: An analysis of primary and secondary prevention trials. Lancet.

[88] Fry A., Littlejohns T.J., Sudlow C., Doherty N., Adamska A., Sprosen T., Collins R., Allen N.E. (2017). Comparison of Sociodemographic and Health-Related Characteristics of UK Biobank Participants with Those of the General Population. Am. J. Epidemiol..

[89] Cohen A.B., Mathews S.C., Dorsey E.R., Bates D.W., Safavi K. (2020). Direct-to-consumer digital health. Lancet Digit. Health.

