# Supplementary material for: Development and Validation of Decision Rules Models to Stratify Coronary Artery Disease, Diabetes, and Hypertension Risk in Preventive Care: Cohort Study of Returning UK Biobank Participants
Source: J Pers Med. 2021 Dec 7;11(12):1322. doi: 10.3390/jpm11121322 (PMC8707007; doi:10.3390/jpm11121322)
Supplement: Supplementary file 1 [file jpm-11-01322-s001.zip › jpm-1416814-supplementary.pdf]

Journal of Personalized Medicine

MDPI

Supplementary Material

Development and validation of decision rules models to stratify coronary artery disease, diabetes, and hypertension risk in preventive care: cohort study of returning UK Biobank participants

Table S1. Search strategy and results.

Module	Search term	Time span	Articles found	Search date
Diabetes	((diabetes [TIAB] AND type 2 [TIAB]) OR impaired glucose [TIAB] OR high blood glucose [TIAB]) AND (biomarker* [TIAB] OR risk [TIAB] OR Hazard [TIAB] OR Odds [TIAB]) AND Meta-analysis [pt] AND 2014:2021 [dp]	2014-20 Oct 2021	1727	20-Oct 2021
Hypertension	(hypertension [TIAB] OR high blood pressure [TIAB]) AND (biomarker* [TIAB] OR risk [TIAB] OR Hazard [TIAB] OR Odds [TIAB]) AND Meta-analysis [pt] AND 2014:2021 [dp]	2014-20 Oct 2021	2073	20-Oct 2021
Coronary artery disease	(atherosclerosis [TIAB] OR plaque build-up [TIAB] OR plaque buildup [TIAB] OR coronary artery disease [TIAB] OR coronary heart disease [TIAB] OR coronary event*	2014-20 Oct 2021	2626	20-Oct-2021

	[TIAB] OR cardiovascular event* [TIAB]) AND (biomarker* [TIAB] OR risk [TIAB] OR Hazard [TIAB] OR Odds [TIAB]) AND Meta-analysis [pt] AND 2014:2021 [dp]			

Figure S1. PRISMA flow for the search for coronary artery disease.

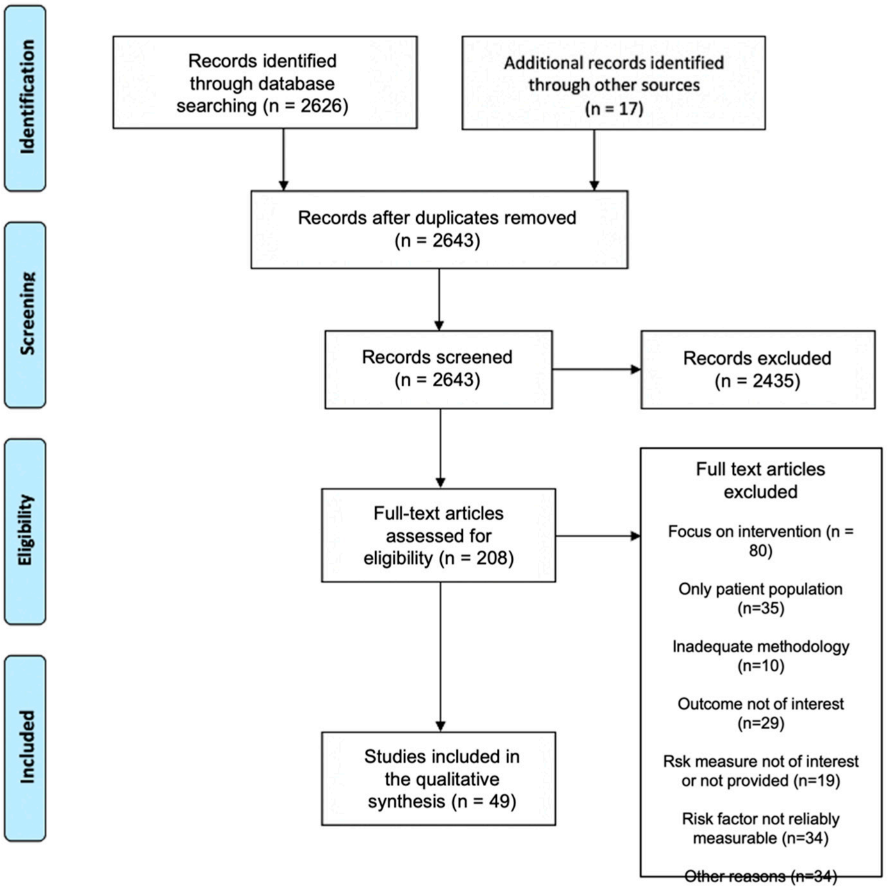

The search resulted in 2626 unique citations, with an additional 17 being identified manually. All of the abstracts were reviewed to see if they met the selection criteria. Then the full text of the preliminary selected articles was reviewed using the selection criteria, which resulted in 208 preliminarily selected articles. The full texts of these were reviewed, resulting in a final identification of 49 relevant publications. The relevant national guideline identified as relevant for this module was the Dutch Society of General Practitioners Guideline for cardiovascular risk management (NHG Cardiovasculair risicomanagement richtlijn). This national guideline was based on the European Society of Cardiology "Guidelines for the management of dyslipidaemias: lipid modification to reduce cardiovascular risk", which were also deemed relevant.

Figure S2. PRISMA flow for the search for hypertension.

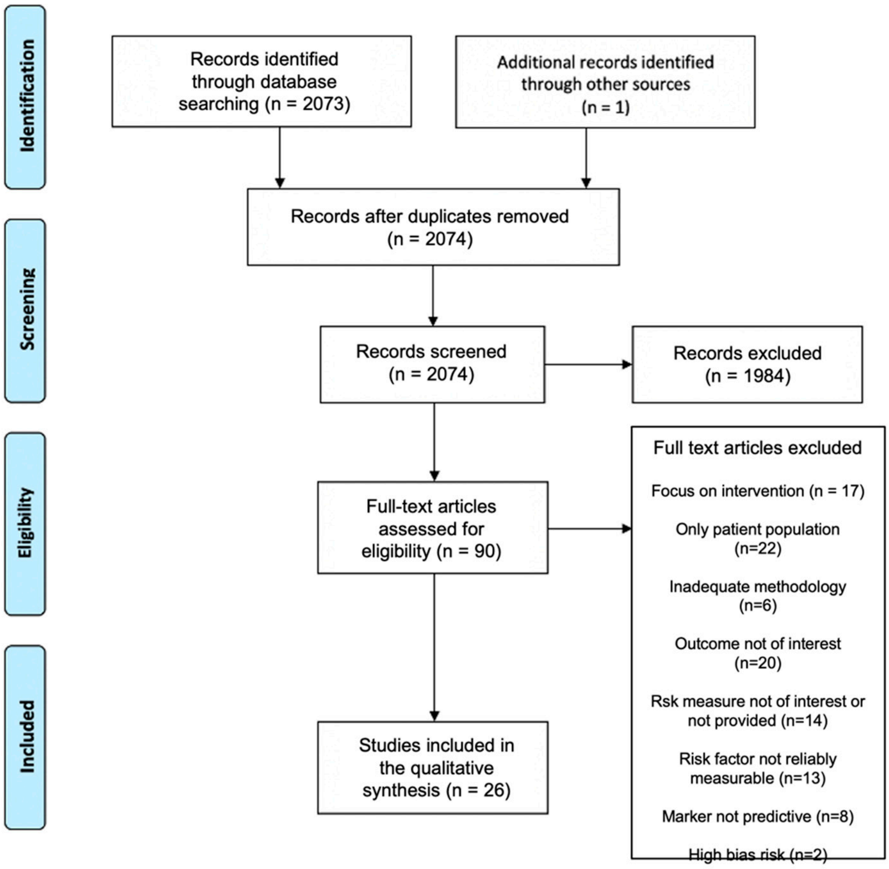

The search resulted in 2073 unique citations, with one additional citation being identified manually. All abstracts were reviewed to see if they met the selection criteria. Then the full text of the preliminary selected articles was reviewed using the selection criteria, which resulted in a final identification of 90 publications. The full texts of 90 preliminarily selected articles were reviewed and resulted in a final identification of 26 relevant publications.

Figure S3. PRISMA flow for the search for diabetes.

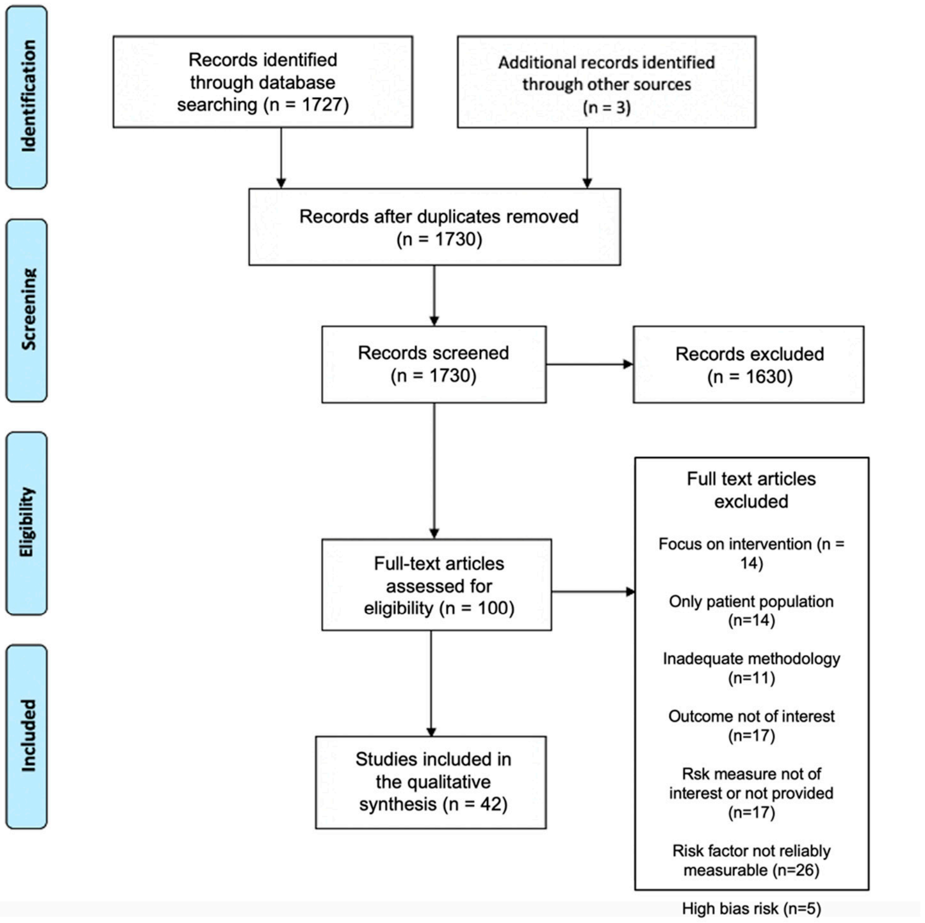

The search resulted in 1727 unique citations, with an additional 3 identified manually. All abstracts were reviewed to see if they met the selection criteria. Then the full text of the preliminary selected articles was reviewed using the selection criteria, which resulted in a final identification of 100 publications. The full texts of 100 preliminarily selected articles were reviewed, resulting in a final identification of 42 relevant publications. The Dutch General Practitioners' association workgroup guideline on type 2 diabetes defines clinical and subclinical thresholds for thresholds for fasting plasma glucose, in accordance with the World Health Organisation/International Diabetes Federation 2006 guideline. These cut-offs of 6.1 mmol/L and 7 mmol/L were taken in the module as "high risk" and "clinical thresholds" respectively.

Table S2. Literature-based risk strata for each health condition.

**Coronary artery disease risk**		
Risk stratum	Variables	Values
High risk	Total cholesterol	> 8 mmol/L
	LDL cholesterol	> 4.9 mmol/L
	Systolic blood pressure	> 180 mmHg
	Clinical risk score<br>Biomarkers other than total cholesterol or LDL	High<br>High
	Clinical risk score<br>PRS	High<br>9th or 10th decile
No elevated risk	Biomarkers<br>Family history<br>Clinical risk score<br>PRS	All withing normal range<br>Negative<br>Low risk<br>< 8th decile
Elevated risk	All other combinations where at least one risk factor is outside normal range	
**Diabetes risk**		
Risk stratum	Variables	Values
High risk	HbA1c	> 6.5%
	Fasting glucose	> 6.1 mmol/L
	HbA1c<br>Weight	5.5% - 6.4%<br>Overweight/obese
	Fasting glucose<br>Weight	5.6 mmol/L - 6.1mmol/L<br>Overweight/Obese
	HbA1c<br>PRS	5.5% - 6.4%<br>9th or 10th decile
	Fasting glucose<br>PRS	5.6 mmol/L - 6.1mmol/L<br>9th or 10th decile
	Clinical risk score	High
	Clinical risk score<br>Age<br>Triglycerides<br>HDL	Elevated<br>> 45<br>> 2.8 mmol/L<br>< 0.9 mmol/L
No elevated risk	Fasting glucose<br>HbA1c<br>Clinical risk score<br>PRS	< 5.6 mmol/L<br>< 5.5%<br>Low risk<br>< 8th decile
Elevated risk	All other combinations where at least one risk factor is outside normal range	
**Hypertension risk**		

Risk stratum	Variables	Values
High risk	Systolic blood pressure	130-140 mmHg
	Diastolic blood pressure	80-90 mmHg
	Clinical risk score	High
	Clinical risk score<br>PRS	Elevated<br>9th or 10th decile
No elevated risk	Systolic blood pressure<br>Diastolic blood pressure<br>Clinical risk score<br>PRS	< 130 mmHg<br>< 80 mmHg<br>Low risk<br>< 8th decile
Elevated risk	All other combinations where at least one risk factor is outside normal range	

Supplementary Methods 1. Polygenic risk score calculation

Polygenic risk scores were created following an additive model for CAD, diabetes and hypertension. To calculate the PGS, the LDpred tool was used following the typical workflow of coordinating the required files, adjustment of SNP weights based on LD and the calculation of the PGS (Vilhjálmsson et al., 2015). The genotyping data and data containing the tested phenotypes outcomes were downloaded from the UKB. All variants with an imputation R2 < 0.4 were removed based on the minimac3 reported R2 readily available in the downloaded genotyping files, using plink (Purcell et al). In total, 3 PGS were calculated for CAD, diabetes, hypertension using summary statistics files referenced in the manuscript. The latter study is a GWAS on blood pressure, which we used to generate PGSs for hypertension. The respective summary statistics files were downloaded and, where necessary, reformatted to be consistent with the format required by LDpred. All variants with a GWAS significance p-value below 0.01 were selected (table S4). SNP ids where converted based on their chromosome, position, ref and alt alleles, to ensure no SNPs were lost due to inconsistent SNP naming between the files.

The Phase 3 genome files were used as an LD reference panel. The overlapping genotypes in the diabetes summary statistics file, the LD reference panel and the UKB genotyping files were extracted and coordinated using the LDpred coordinate option. This was followed by a reweighing step for the SNPs based on their LD. LDpred-inf option of LDpred version 1.0.11 was used to calculate the PGS scores where the causal fraction -f was set to 1, the LD region -ldf 1000 as suggested in their guidelines. The calculations of the PGS scores described above were conducted on a per chromosome basis. Afterward, the scores for all chromosomes were summed resulting in 1 PGS file containing a PGS for each individual. Next, we calculated the odds ratios between the 10% individuals with the highest risk against the rest of the population. We have conducted this analysis with and without adjustment for genotyping array, first 4 principal components, age and sex. We determined the risk increase that can be derived from solely genetic data and compared this to the risk increase predicted based on genetic data, age and sex (table S5). These results are in line with those reported by others (fig S4).

Table S3. Summary statistics data: The column denotes the phenotype the PGS was used for. Followed by the number of cases and controls used in the GWAS study. The last three columns describe the total number of SNPs in the summary statistic file, those with a p-value <0.01 and those overlapping with the ones present in the LD reference panel and the UKB genotyping chip.

Phenotype	Cases	Controls	SNPs	p<0.01	Overlapping
T2D	26676	132532	12056346	199120	159290
CAD	60801	123504	9455805	139885	105325
Hypertension	80792	99785	28276170	400016	243528

T2D = type 2 diabetes, CAD = coronary artery disease, SNP = single nucleotide polymorphism.

Table S4. UKB columns used to calculate disease incidence.

Disease	Column(s)	Data-Field(s)	Value
Diabetes	Diabetes diagnosed by doctor	2443	yes
	Non-cancer illness code, self-reported	20002	type 2 diabetes
	ICD10	41270, 41202, 41204, 40001, 40002	E11 and related sub-codes
Hypertension	Vascular/heart problems diagnosed by doctor	6150	high blood pressure
	Non-cancer illness code, self-reported	20002	hypertension
	Medication for cholesterol, blood pressure or diabetes	6177	blood pressure medication
	ICD10	41270, 41202, 41204, 40001, 40002	E10
CAD	Vascular/heart problems diagnosed by doctor	6150	heart attack
	Non-cancer illness code, self-reported	20002	heart attack
	Operation code	20004	PTCA, CABG, triple heart bypass
	ICD9	41271, 41203, 41205	410-412
	ICD10	41270, 41202, 41204, 40001, 40002	I21-24, I25.2
	OPCS4	41272, 41200, 41210	K40-46, K50.1, K75

Figure S4. Selection process patient population for the analyses for different health conditions.

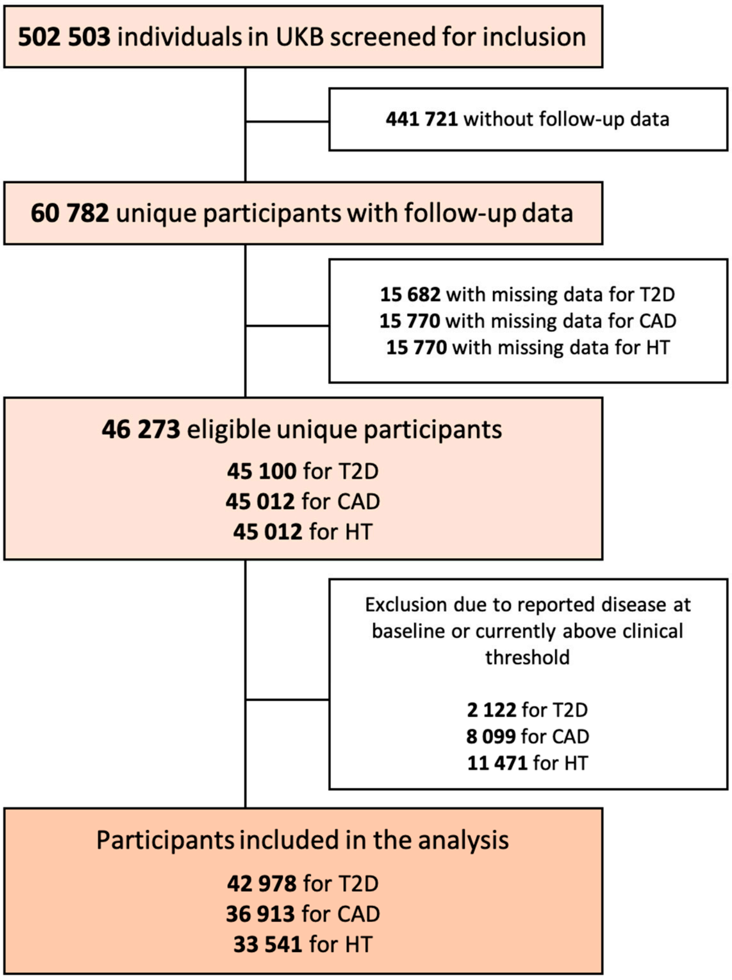

Table S5. Risk for the individuals in the highest risk decile for each genetic risk score, compared to rest of population for the complete UKB population (n=442 687). Second and third column show hazard ratios calculated based on a logistic regression model adjusted for the respective variables. In all cases the difference with the remainder of the population was statistically significant (p-value < E-100).

	**Unadjusted PRS**	**PRS adjusted for 4 PCs and array type**	**PRS adjusted for 4 PCs, array type, sex and age**	**Age and sex**
**CAD**	1.99 (1.93-2.04)	2.06 (2-2.11)	4.77 (4.68-4.86)	3.74 (3.66-3.82)
**T2D**	1.93 (1.88-1.98)	2.05 (2-2.09)	2.90 (2.84-2.96)	2.22 (2.17-2.28)
**Hypertension**	1.37 (1.35-1.39)	1.38 (1.37-1.4)	1.85 (1.83-1.87)	1.66 (1.64-1.68)

Table S6. Sensitivity analysis for all models. Sensitivity, specificity, positive predictive value (PPV), and negative predictive (NPV) value all reported with 95% confidence interval.

**Model and health condition**	**Sensitivity**	**Specificity**	**PPV**	**NPV**
**CAD**				
FRS women	81.8% (73.8%-88.2%)	57.0% (56.2%- 57.9%)	1.7% (1.5%-2.0%)	99.6% (99.5%-99.7%)
FRS men	75.0% (68.7%-80.6%)	54.5% (53.5%-55.5%)	3.7% (3.5%-4.0%)	98.9% (98.7%-99.2%)
Rule model	72% (68%-76%)	59.8% (59.3%-60.3%)	2.5% (2.4%-2.6%)	99.4% (99.3%-99.5%)
**T2D**				
FRS	72.2% (69.4%-75.0%)	71.6% (71.2%-72.1%)	5.7% (3.3%-3.5%)	99.1% (99.0%-99.2%)
Rule model	81.5% (79.1%-84.0%)	68.2% (67.8%-68.6%)	5.8% (5.6%-6%)	99.4% (99.3%-99.4%)
**Hypertension**				
FRS	97.4% (96.7%-98.0%)	22.1% (21.7%-22.6%)	8.7% (8.6%-8.8%)	99.1% (98.9%-99.3%)
Rule model	73.9% (72.7%-75.7%)	65.5% (65.0%- 66.0%)	14.1% (13.7%-14.4%)	97.1% (96.9%-97.2%)

Figure S5. Prevalence of disease of individuals classed into different risked strata based on their genetic susceptibility scores.

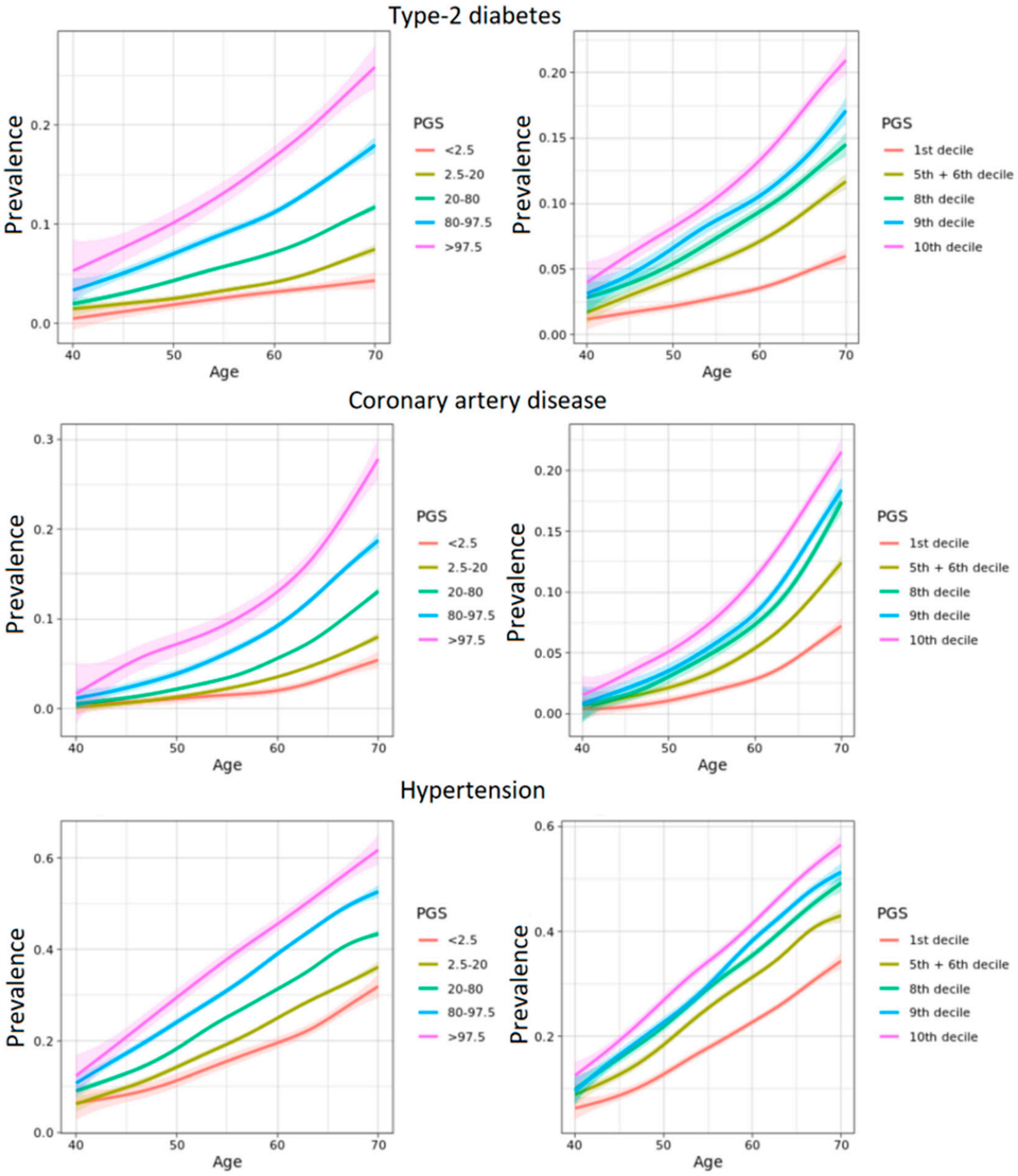

Left side shows risk strata as defined in Mars et al. Scores were corrected for first 4 principal components and array type. Only individuals with follow-up data are included (table 3). The AUROCs reported in table 2 pertain to the comparison between the 1-7th deciles and the 10th decile (table 2)._
